# Bioactive Yoghurt Containing Curcumin and Chlorogenic Acid Reduces Inflammation in Postmenopausal Women

**DOI:** 10.3390/nu14214619

**Published:** 2022-11-02

**Authors:** Noha Ahmed Nasef, Rohith N. Thota, Anthony N. Mutukumira, Kay Rutherfurd-Markwick, Martin Dickens, Pramod Gopal, Harjinder Singh, Manohar L. Garg

**Affiliations:** 1 Riddet Research Institute, Massey University, Palmerston North 4442, New Zealand; 2 Macquarie Medical School, Macquarie University, North Ryde, NSW 2109, Australia; 3 Nutraceuticals Research Program, School of Biomedical Sciences and Pharmacy, University of Newcastle, Callaghan, NSW 2308, Australia; 4 School of Food and Advanced Technology, College of Science, Massey University, Auckland 0745, New Zealand; 5 School of Health Sciences, College of Health, Massey University, Auckland 0745, New Zealand; 6 New Zealand Institute for Plant and Food Research, Palmerston North 4442, New Zealand

**Keywords:** menopause, inflammation, yoghurt, curcumin, chlorogenic acid, functional food

## Abstract

Menopause is marked by a gradual and permanent decrease of estrogen from the ovaries, leading to metabolic and physiological changes in the body. Combined with increased body mass index, postmenopausal women have elevated systemic inflammation and metabolic disturbances leading to increased risk of developing chronic diseases. A bioactive coconut yoghurt containing curcumin and chlorogenic acid was developed with the potential to target inflammatory processes. In this randomized crossover study, healthy postmenopausal women with a BMI of 25–40 were recruited to consume 125 g of either the bioactive or placebo yoghurt. Blood samples were collected at baseline, 30 min, and 1, 2, 3 and 4 h postprandially. Plasma inflammatory markers (TNFα and IL6) and metabolic markers (triglycerides, insulin and glucose) were measured. Participants had significantly lower plasma TNFα C_max_ after consumption of the bioactive yoghurt compared to placebo (mean difference = 0.3 pg/mL; *p* = 0.04). Additionally, plasma TNFα was significantly lower postprandially compared to baseline after consumption of the bioactive yogurt but not the placebo. No differences were observed in the metabolic markers measured. Conclusions: The bioactive yoghurt fortified with curcumin and chlorogenic acid has the potential to reduce inflammatory mediators; however, a larger and longer-term study is required to confirm these findings.

## 1. Introduction

Menopause is a significant phase in a woman’s life, marked by the final episodes of menstrual bleeding, that is associated with permanent decline in ovarian function. The decline in ovarian function results in hormone deficiency, in particular estrogen, that contributes to increased incidence of osteoporosis, cardiovascular diseases, metabolic disorders and cognitive deterioration [1,2]. Reduction in circulating estrogen after menopause results in a number of changes in the body including metabolic disturbances and increased body fat [3]. Moreover, postmenopausal women are more prone to impaired glucose, insulin and triglyceride response, after eating a meal (known as the postprandial state), compared with premenopausal women [4,5,6].

Chronic elevation in postprandial glucose, insulin and triglycerides in the blood has detrimental effects on regulation of metabolic organs such as the liver, pancreas and muscles [7,8,9]. It is hypothesized that sustained concentrations of postprandial glucose and fat increase oxidative stress and inflammation, eventually leading to metabolic diseases such as type 2 diabetes and cardiovascular disease [8,10]. Given that humans spend the majority of each day in a postprandial state, interventions that regulate the postprandial response warrant investigation.

After menopause, adipose tissue becomes the primary site for estrogen production [11] and, in combination with metabolic changes, postmenopausal women are prone to become overweight and obese which leads to further modifications in metabolism, alteration to the lipid profile and increased inflammation [12,13,14]. Obesity is associated with gradual adipose tissue infiltration with macrophages that secrete proinflammatory cytokines such as interleukin-6 (IL6) and tumor necrosis factor-α (TNFα) [15]. Therefore, in combination with increased body mass index (BMI), postmenopausal women were shown to have elevated levels of these systemic markers of inflammation [16,17,18]. A long-term uncontrolled, inflammatory response combined with metabolic disturbances in postmenopausal women increases their risk of developing chronic conditions including cancer [11,19], cardiovascular disease [20,21] and type 2 diabetes [22].

There is growing interest in alternative natural treatments to control inflammation and metabolic disorders, that can be a regular part of daily diets [23]. In particular, functional foods are aimed at targeting physiological processes involved in chronic non-communicable diseases [24]. An example of a promising naturally-derived anti-inflammatory compound suitable for incorporation into functional foods is curcumin.

Curcumin has been extensively studied for its anti-inflammatory and anti-diabetic health benefits [25]. We have previously shown that curcumin effectively reduced postprandial glucose in healthy participants [26]. Additionally, curcumin is known to block the action and production of TNFα [27,28]. However, curcumin has low bioavailability and solubility [29,30].

We have previously shown in vitro, that co-administration of curcumin with another naturally-derived compound, chlorogenic acid (CGA), works synergistically to reduce inflammation in a macrophage cell line [31]. CGA is obtained primarily from coffee and has potent antioxidant properties [32]. In the study, the macrophage cell line was stimulated with lipopolysaccharides (LPS) to induce inflammation. When the LPS-stimulated cells were treated with curcumin in combination with CGA for 4 h, gene expression of several inflammatory markers, including TNFα and IL6, was significantly decreased compared to when the cell line was treated with curcumin or CGA alone [31]. We have further developed a stable functional yoghurt with acceptable organoleptic properties that contains curcumin and CGA, with the aim of targeting inflammatory and metabolic processes.

Here, we investigate the acute effects of consuming this bioactive yoghurt on postprandial markers of inflammation (TNFα and IL6) and metabolism (triglycerides, insulin and glucose) in overweight or obese postmenopausal women.

## 2. Materials and Methods

### 2.1. Study Design

**Figure 1:**
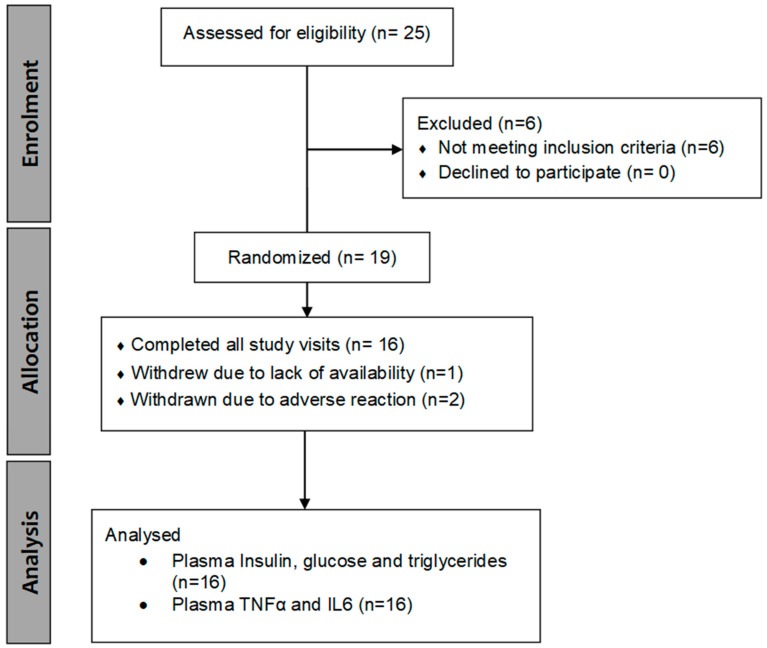
CONSORT flow diagram.

Healthy female participants (n = 16, see Section 2.2 for sample size calculation) were recruited for this randomized, double-blind, placebo-controlled, crossover study (refer to CONSORT flow diagram, Figure 1). Ethical approval for the study was obtained from the Health and Disability Ethics Committee (Ethics ref: 20/STH/96). Informed consent was obtained from the participants in this study and the trial was registered with the Australia New Zealand Clinical Trial Registry (ANZCTR ID: ACTRN12620001002976). Participants were recruited from October 2020 to April 2021 through noticeboards, and email lists at Massey University, Palmerston North, New Zealand.

Inclusion criteria included women aged between 45 and 65 with a BMI of 25–40 kg/m^2^ who had been menopausal for at least a year (defined as 12 months of consecutive amenorrhea). Exclusion criteria included taking hormone replacement therapy; suffering from cancer, HIV/Aids or inflammatory bowel disease; history of congestive heart failure, stroke, or cardiovascular disease; having a history of gastrointestinal disorder or liver disease.

Eligible participants were randomized to attend two trial visits, each separated by at least 1-week of washout period. During their first study visit, all participants completed a medical and physical activity questionnaire and their height, weight and blood pressure were recorded. The participants were randomly allocated to one of two pre-generated trial sequences where they either received the placebo yoghurt first or the bioactive yoghurt first. Invited participants came fasted to the Human Clinical Research Unit of Plant & Food Research, Palmerston North for their scheduled trial visit and were cannulated in their forearm. A baseline blood sample was then collected from the canula. Following this, participants were instructed to consume a breakfast consisting of a breakfast bar (Uncle Tobys Breakfast Bakes Apple & Cinnamon, supplementary Figure S1), 250 mL of water and 125 g of the study yoghurt (macronutrient content of the meal can be found in Table 1). Following this, blood samples were collected after 30 min, and 1, 2, 3 and 4 h postprandially. Participants were requested to drink 250 mL of water every hour, to maintain hydration during their visit, and remain in a resting state. After a minimum of a one week wash-out period, the participants were crossed over to the second treatment. The collected blood was centrifuged at 18–25 °C at 1300× *g* for 10 min and plasma collected. The supernatant plasma was sent to the Nutrition Laboratory, Massey University, Palmerston North, New Zealand for analyses of triglycerides (GPO-PAP), glucose (GOD-PAP), insulin (Radioimmunoassay), TNFα and IL6 (Milliplex Magnetic Bead Panel, Bioplex analyzer. Kit #HMHEMAG-34K).

### 2.2. Study Intervention Yoghurts

The yoghurts for the study were prepared in a food accredited lab. They contained Kara coconut, coconut sugar, and culture (see details below). The bioactive yoghurt also contained curcumin and CGA. All materials used in the preparation of the yoghurt were food-grade quality. The formulation of the yoghurt used in this study is shown in Table 2. Ultra-high temperature (UHT)-processed coconut cream (2455.51 g; Kara^TM^, PT Pulau Sambu, Indonesia; supplementary Table S2) was placed on top of a boiler pot containing water heated to 90 °C and the cream was stirred every 5 min. Organic coconut sugar (21.018 g; Matsanta Foods Ltd., New Zealand; supplementary Figure S1) purchased from a local supermarket was added into the heated coconut cream (50 ± 1 °C). The mixture was then heated to 90 ± 3 °C and held at this temperature for 5 min. Following this, the mixture was allowed to cool to 50 ± 2 °C in a water bath at ambient temperature (20 °C). The heat-treated coconut cream was divided into two portions: 1184.58 g of the coconut cream was used to make yoghurt containing bioactives and the reminder was the control (placebo).

The coconut yoghurt containing bioactives was made by adding coffee extract stage 2 (Coffee Brewmaster Ltd., Williamstown, Australia) and Curcumin C3 complex AU powder (Sami Labs Ltd., Woolloomooloo, Australia) to the coconut cream mixture and mixed using a flat beater (KitchenAid K5SS, St Joseph, MI, USA) set to a low speed for 5 min. Starter culture (VEGE 022 LYO 200 DCU, Danisco, Saint-Marcellin, France) was inoculated into the coconut cream mixture and mixed for 15 min at low speed. 125 g of the mixture was then added into food grade glass jars. The glass jars containing inoculated coconut cream mixtures were incubated in a water bath (Grant GLS400, Essex, UK) set at 43 ± 1 °C until pH 4.3 ± 0.2 was achieved as per the yoghurt requirements in the Australian New Zealand Food Standards Code (2015)—Standard 2.5.3. The placebo was prepared in the same way, but without the addition of bioactives. After fermentation, the yoghurt samples were stored overnight at 4 °C ± 1°C to strengthen the gel. The constituent cultures (*Lactobacillus bulgaricus* and *Streptococcus thermophilus*) were analysed at the beginning (day 1) and end of storage (day 14) during the shelf-life of the yoghurt. To ensure the safe consumption of the yoghurt made for the human study, the yoghurt samples were tested for presence of coliforms, *Escherichia coli* and *Staphylococcus aureus*, using standard microbiological methods (supplementary Section S1). The food safety of the yoghurt was assessed and approved by the Plant & Food Research food safety committee for consumption by the participants.

### 2.3. Statistical Analysis

All analysis was performed using GraphPad prism version 9.2.0. Postprandial data after consumption of the bioactive and placebo yoghurts were compared using a mixed-effects model with the Geisser–Greenhouse correction combined with either Šídák’s multiple comparisons (TNFα and IL6) or Dunnett’s multiple comparisons (triglycerides, insulin and glucose) post-hoc tests. The area under the curve (AUC) was calculated from hourly measurements using the trapezoid rule. Plasma C_max_ was compared between the yoghurts using a paired two-tailed *t*-test. Pre- and post yoghurt consumption for both yoghurts was analyzed using a repeated measures one-way ANOVA combined with Holm–Šídák’s multiple comparisons post-hoc test. Statistical significance was accepted at *p* < 0.05. Participant characteristics are presented as median and interquartile range (IQR). Study data are presented as mean ± standard error of the mean (SEM) or standard deviation (SD).

A paired *t*-test (PS sample size and power software [33]) was used for calculating sample size. The sample size calculation was based on an anticipated 1.23 pg/mL [28] difference in TNF-α concentrations between the treatment groups with level of significance 0.05 and 80% power. Using a standard deviation of 1.5 pg/mL in TNFα, a minimum of 14 subjects would be required in each interventional treatment. To allow for dropouts and non-compliant subjects, 19 participants were recruited.

## 3. Results

### 3.1. Participant Characterisitics

Sixteen participants completed the study (CONSORT diagram; Figure 1, Table 3). Participants had a median age of 58 (IQR, 55 to 61), a median BMI of 27 (IQR 25 to 35) [34] and their median last menstural cycle was 7 years before their first study visit (IQR, 3 to 19). The women were mostly moderately active (Table 3) with a median physical activity score of 2541 (IQR, 1239 to 3728) MET equivalent/min and a physical activity score of 2 (moderately active). Homeostasis model assessment–estimated insulin resistance (HOMA-IR) was measured from fasting plasma insulin and glucose collected at baseline, as a measure of insulin resistance (Table 3). Median HOMA-IR was 2.1 (IQR, 1.8 to 2.7), suggesting a higher risk of insulin resistance in some of the participants (cut-off HOMA-IR values for risk of metabolic syndrome in different populations range between 1.5 and 3.8 [35]).

HOMA-IR = homeostasis model assessment–estimated insulin resistance: [Fasting insulin (µU/L) × fasting glucose (nmol/L)]/22.5.

### 3.2. Influence of Study Yoghurt on Inflammatory Markers

Participants consumed either a bioactive or placebo yoghurt, and plasma TNFα and IL6 were measured postprandially as markers of inflammation.

**Figure 2:**
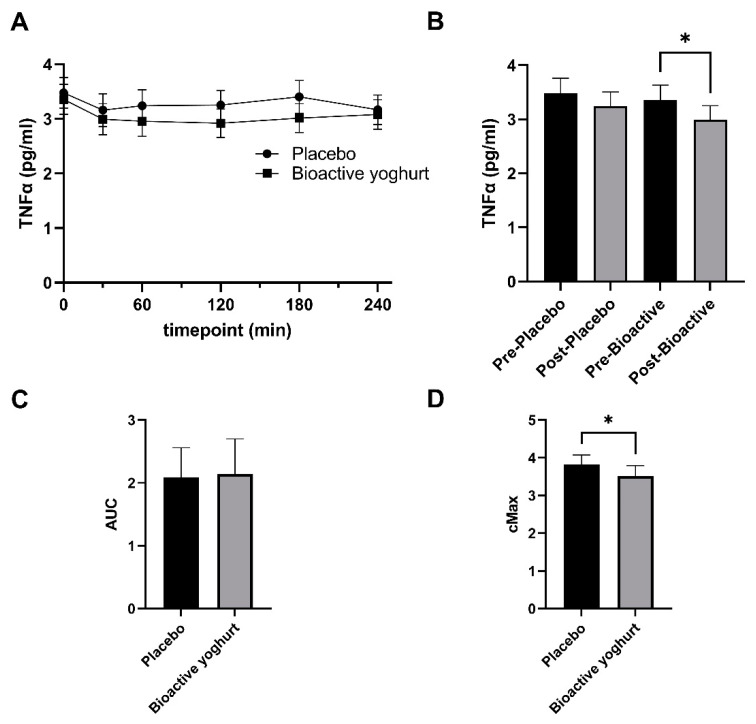
Plasma TNFα. Plasma was collected from participants at baseline and after yoghurt consumption and measured for TNFα. TNFα represented as (**A**) Timepoints (**B**) before and after yoghurt consumption (**C**) AUC and (**D**) Maximum concentration (C_max_). Data represented as mean and SEM. * represents statistical significance (*p* < 0.05). n = 16.

The plasma TNFα ranged from 2.923 to 3.357 pg/mL at any given time (Figure 2A). The mean plasma TNFα for the placebo yoghurt was 3.285 (± 0.13 SD) and for the bioactive yoghurt was 3.055 (±0.16 SD) pg/mL. There was a slight dip in plasma TNFα after yoghurt consumption (Figure 2A,B). However, time was not a significant factor for plasma TNFα (two-way ANOVA; *p* = 0.1017). On the other hand, when plasma TNFα was combined and averaged for all post-yoghurt consumption timepoints and compared with baseline levels, participants had significantly lower plasma TNFα after consumption of the bioactive yoghurt (*p* = 0.0326) but not the placebo yoghurt (*p* = 0.1003) (Figure 2B). Furthermore, the maximum concentration of plasma TNFα (C_max_) was significantly lower after participants consumed the bioactive yoghurt compared to placebo (Figure 2D; mean difference = 0.3 pg/mL; *p* = 0.04). No significant difference in AUC was seen between the two yoghurts (Figure 2C).

Only eight participants had detectable plasma IL6 at all timepoints (supplementary Table S1). There was large inter-individual variability in the plasma IL6 which ranged from 3.740 pg/mL to 140.9 pg/mL. Furthermore, there was no significant difference observed between plasma IL6 after consumption of placebo and bioactive yoghurt (supplementary Section S2).

### 3.3. Study Yoghurts and Metabolic Markers

**Figure 3:**
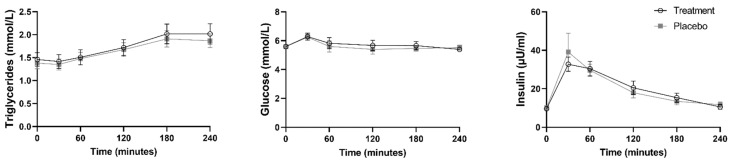
Plasma metabolic markers before and after consumption of study yoghurts. Data represented as mean and SEM.

Plasma triglycerides, glucose and insulin were also measured in blood collected from the participants (Figure 3).

Participants had an average fasting plasma triglyceride of 1.4 mmol/L. Plasma triglycerides significantly increased from baseline after consumption of the placebo yoghurt at 1 h post-consumption (mean difference = 0.09823 mmol/L; *p* = 0.0441) and continued to be significantly higher than baseline until the final measured timepoint at 4 h post-yoghurt consumption (mean difference = 0.4863 mmol/L; *p* = 0.0001). Similarly, after consumption of the bioactive yoghurt, plasma triglycerides significantly increased from baseline to 2 h after yoghurt consumption (mean difference = 0.2594 mmol/L; *p* = 0.0021) and continued to be significantly higher than baseline until the final measured timepoint at 4 h (mean difference = 0.5573 mmol/L; *p* = 0.0004). No significant difference was observed in plasma triglycerides between the placebo and bioactive yoghurt.

Participants had an average fasting plasma glucose of 5.6 mmol/L. Plasma glucose significantly increased from baseline after consumption of the placebo yoghurt (mean difference = 0.6501 mmol/L; *p* = 0.0009) and bioactive yoghurt (mean difference = 0.6908 mmol/L; *p* = 0.0069) within 30 min of participants eating the yoghurts. Following this, plasma glucose returned back to baseline levels at 1 h after consumption of both yoghurts. There was no significant difference in plasma glucose between the placebo and bioactive yoghurt.

Participants had an average fasting plasma insulin of 9.9 µU/mL. Plasma insulin significantly increased from baseline to 30 min after consumption of the placebo yoghurt (mean difference = 28.98 µU/mL; *p* = 0.0314) and bioactive yoghurt (mean difference = 23.13 µU/mL; *p* < 0.0001). Plasma insulin continued to be significantly higher than baseline until 3 h post consumption of the placebo (mean difference = 3.367; *p* = 0.0050) and bioactive (mean difference = 5.755; *p* = 0.0202) yoghurts. At 4 h post yoghurt consumption, plasma insulin returned back to baseline. No difference in plasma insulin was observed between the placebo and bioactive yoghurt.

## 4. Discussion

In this study, we evaluated the acute effects of a bioactive yoghurt containing curcumin and CGA on inflammatory and metabolic markers in women with increased risk of inflammation and metabolic disorders. For the study, women consumed 125 g of yoghurt and their markers were measured postprandially. Despite the small amount of yoghurt consumed by the participants and the acute nature of the study, we observed an impact of the bioactive yoghurt on inflammatory marker TNFα.

Elevated plasma triglycerides and glucose after consumption of a high-fat meal (≥30 g), increase systemic inflammation [36,37] which may be exacerbated in states of metabolic dysfunction including post-menopause, overweight and obesity. Thus, targeting postprandial inflammation may disrupt the association between frequent postprandial lipemia and glycemia and progression to metabolic disease such as cardiovascular disease and type 2 diabetes [8,10]. The meal given to the participants in this study contained 40 g of fat which significantly elevated plasma triglycerides in the participants after consumption. With the elevated plasma triglycerides due to fat consumption (≥30 g) [37] combined with an overweight state and estrogen deficiency, it is likely that the plasma TNFα changes that occurred in the study are related to postprandial inflammation. However, the type of fat that is consumed influences the postprandial response [37]. Despite the participants consuming 40 g of mostly saturated fat, where 75% of the fat was from the yoghurt, TNFα changes were subtle, suggesting that coconut cream would be a suitable ingredient for creating a functional food with acceptable sensory attributes, for postmenopausal women.

TNFα is a pleiotropic inflammatory cytokine involved in a variety of biological processes and promotes acute inflammatory responses including oxidative stress. Additionally, TNFα is one of the major indicators of chronic inflammation, thus blockers of TNFα such as monoclonal antibodies have been developed [27]. Naturally-derived food products that target TNFα and can be consumed as part of the daily diet would therefore be appealing as a means to reduce the risk of chronic inflammation.

TNFα has been associated with several diseases and dysfunction in postmenopausal women. Inhibition of TNFα was shown to be effective against vascular dysfunction particularly in aging [38] and postmenopausal women [39]. Furthermore, studies show that TNFα may be involved as a low-grade stimulus of osteoporosis, insulin resistance, and atherogenesis in postmenopausal women which is related to estrogen deficiency [40]. It is known that TNFα directly impairs insulin signaling through inhibition of tyrosine kinase activity of the insulin receptor and thus could be a critical mechanism whereby adiposity induces peripheral insulin resistance [41]. However, in the current study we did not measure insulin resistance directly and the participants did not suffer from type 2 diabetes. Therefore, the impact of plasma TNFα measured in the study on insulin resistance is unknown. Long-term studies are required to further understand the impact of inhibiting postprandial plasma TNFα after bioactive yoghurt consumption, and its effects on vascular dysfunction and insulin resistance.

Previous studies including postmenopausal women with a BMI of 25 or over have reported the average plasma TNFα ranged from 1.0 to 7.41 pg/mL [39,42,43,44,45]. This was consistent with our findings where the plasma TNFα ranged from 2.923 to 3.357 pg/mL. In our study, we found a transient decrease in postprandial plasma TNFα starting from 30 min after consumption of both yoghurts. This transient decrease has been seen in previous studies after consumption of a high fat meal and is associated with postprandial insulin and its potential anti-inflammatory effects [46,47]. Interestingly, this transient decrease has been mainly shown to be significant in healthy participants that do not have insulin resistance [48,49,50]. The prevalence of insulin resistance increases from pre to post-menopause [51]. In the present study, HOMA-IR scores signaled possible insulin resistance in some of the participants. In line with this, we only observed a significant decrease in postprandial TNFα after consumption of the bioactive yoghurt but not the control yoghurt in this study. These findings, together with observations from previous studies, suggest that the bioactive compounds in the yoghurt may contribute to modulation of insulin sensitivity through a reduction in postprandial TNFα, in postmenopausal women. However, longer-term studies are required to further evaluate the effect of the bioactive yoghurt on postprandial TNFα, inflammation and insulin sensitivity.

We previously reported that curcumin and CGA, in combination, reduced *TNFα* and *IL6* gene expression in a macrophage cell line [31]. Several studies have reported that curcumin affects inflammatory markers including TNFα and IL6 [27,31,52,53]. Curcumin’s inhibition of TNFα has been studied extensively in vitro [52]. Inhibition of TNFα by curcumin primarily occurs at the transcriptional level in many cell types via multiple inflammatory pathways, including downregulation of nuclear factor kappa B (NFκB), a master regulator of inflammation. Provided that curcumin can reach these cells, it is likely that we would observe an anti-inflammatory effect in humans. A systematic review and meta-analysis of randomized control trials indicated that curcumin could significantly reduce plasma TNFα concentration [27]. These trials used curcumin as a nutraceutical supplement for at least 1 month. Furthermore, in some of the trials analyzed in the review, curcumin was combined with another bioactive compound (such as piperine), in order to overcome its low bioavailability [54,55]. In our present study, we combined curcumin with an antioxidant (CGA) and show that consuming curcumin in a functional food can also reduce plasma TNFα, within hours of consumption.

In the current study, another marker of inflammation, plasma IL6, was also measured. Elevated levels of fasting plasma IL6 has been linked with increased risk of cardiovascular disease [20,21] and type 2 diabetes [22] in postmenopausal women. The fasting plasma IL6 levels in the current study ranged from 7.1 pg/mL to 130 pg/mL for the eight participants who had detectable levels of plasma IL6. The fasting IL6 levels in this study suggests that some of the study participants are at a higher risk for developing type 2 diabetes [22]. However, due to interindividual variability, it was not possible to make any conclusions regarding the impact of the bioactive yoghurt on postprandial IL6.

We did not observe any differences between the placebo and bioactive yoghurt for any of the metabolic markers measured in the participants. We have previously shown that curcumin effectively reduced postprandial glucose in healthy participants [26]. However, in the previous study, participants consumed higher amounts of simple carbohydrate, consisting of two slices of white toast and 250 mL chocolate flavored drink, providing a total of 56 g of carbohydrate [26], compared to the current study that had carbohydrate from an oat breakfast bar and coconut cream and sugar, providing 37.51 g of carbohydrate. This was also reflected in the change in plasma glucose from baseline, where the previous study showed a plasma glucose increase of 1.5–2 mmol/L from baseline to 30 min post-consumption [26] compared to only an average plasma glucose increase of 0.6 mmol/L in the current study. A higher sugar dose may have been required to see an effect. Furthermore, the oats and coconut sugar may have dampened the glycemic response in the present study. Additionally, in the previous study, participants consumed curcumin in the more traditional form (commercial tablets), providing 180 mg of curcumin, compared to the current study, where participants consumed a yoghurt containing 103 mg of curcumin powder.

Apart from a potentially high HOMA-IR score in some of the participants, fasting metabolic markers were within the normal range, suggesting that the participants were healthy. Therefore, plasma glucose, triglycerides and insulin concentrations may not have been abnormally high enough to be altered by the bioactive compounds. As expected, plasma triglycerides, insulin and glucose significantly increased after consumption of both yoghurts, due to the presence of fat and sugar. However, a larger sample size and longer-term studies in individuals with more pronounced metabolic aberrations are required to identify any differences between the placebo and bioactive yoghurt in these markers after consumption.

Both the placebo and bioactive food in this study were made of coconut yoghurt. Coconut milk, which is the base of the yoghurt, is the liquid extracted from the shredded meat of matured coconut, and is composed predominantly of medium-chain fatty acids (MCFA). MCFA are known to have health benefits, including improving metabolic function [22]. Furthermore, the yoghurts used in this study contained live probiotic cultures, which also have health benefits in relation to immune and digestive health [56].

Both the placebo and bioactive food in this study were made of coconut yoghurt. It is therefore possible that the yoghurt base itself influenced the metabolic markers measured in this study and therefore masked some of the effects from the bioactive compounds.

When working with a functional food base such as yoghurt, it is necessary to factor in taste and consistency and stability of the ingredients combined. These considerations dictate and limit the amount of bioactive compounds that can be incorporated into the functional food. It is likely that a higher dose of the bioactive compounds would have produced a more pronounced effect on the inflammatory and metabolic markers. However, in a real-world scenario, to achieve benefit, uptake of the food is as important as the nutrient profile.

## 5. Conclusions

Functional foods offer a promising opportunity to incorporate healthy foods into the daily diet to improve health outcomes. In this study we developed a functional yoghurt with the bioactive compounds curcumin and CGA. The functional yoghurt was shown to reduce TNFα, an inflammatory cytokine targeted for inhibition by many pharmaceuticals and nutraceuticals in various chronic inflammatory diseases. While the study highlighted potentially acute beneficial effects, the long-term effects of consuming the yoghurt are unknown. It is also not known whether the synergistic effects we observed in vitro, between CGA and curcumin, are reflected in the human study. Further studies are required to better understand the impact of regular consumption of this yoghurt and the mechanisms of action involved.

## Figures and Tables

**Table 1:** Macronutrient content of study meal. Details of the yoghurt formulation in Section 2.2.

	Yoghurt			
Macronutrient	Coconut Cream	Coconut Sugar	Breakfast Bar	Total
Fat (g)	30.5	0.012	9.6	40.11
-Saturated fat (g)	28.9	0.012	1.1	30.01
Carbohydrate (g)	2.6	1.007	33.9	37.51
-Sugar (g)	2.6	0.9116	7.5	11.01
Fibre (g)	0	0.0106	7.3	7.31

**Table 2:** Formulation of coconut cream yoghurt with added bioactives.

	Bioactive		Placebo	
Ingredient	g	%	g	%
Pasteurized Kara™ coconut and coconut sugar	1184.58	99.818	1270.9	100
Coffee extract *	1.17	0.099	0	0
Curcumin C^3^ complex	0.99	0.083	0	0
Fermentation culture	0.027	0.0023	0.027	0.0021

* Contains 2.61 mg CGA per gram of coffee extract. A 125 g bioactive yoghurt contained 0.32 mg CGA and 103 mg curcumin C^3^.

**Table 3:** Baseline characteristics of the study participants. Data represented as median and interquartile range.

Characteristic	Median (IQR)
n	16
Age (years)	58 (55 to 61)
Last menstrual cycle (years) *	7 (3 to 19)
Height (m)	1.7 (1.6 to 1.7)
Weight (Kg)	76 (72 to 94)
BMI	27 (25 to 35)
Systolic	126 (109 to 140)
Diastolic	84 (74 to 93)
Physical activity (MET-equivalent/min)	2541 (1239 to 3728)
Physical activity category	2 (2 to 3)
HOMA-IR	2.1 (1.8 to 2.7)

* Fourteen participants provided a specific date. The remaining two participants could not remember and therefore were not included in this dataset. MET = metabolic equivalent of task. Physical activity category 1 = low activity; 2 = moderately active; 3 = highly active.

## Data Availability

Plasma TNFα data available upon request subject to ethics approval of retrospective data analysis.

## Supplementary Materials

The following supporting information can be downloaded at: https://www.mdpi.com/article/10.3390/nu14214619/s1, Section S1: Yoghurt testing for pathogens and total aerobic counts after fermentation; Section S2: IL6 data; Table S1: Plasma IL6 (pg/mL) at baseline and different timepoint (min) after yoghurt consumption; Figure S1: Uncle toby’s breakfast bar nutrient label extracted from countdown NZ online website (11 October 2022); Figure S2: Coconut sugar nutrient label from Matakana online store (11 October 2022); Table S2: Kara UHT Natural Coconut Cream nutrient summary from Foodworks 10 Version: 10.0.4266.
