# Supplementary material for: Bioactive Yoghurt Containing Curcumin and Chlorogenic Acid Reduces Inflammation in Postmenopausal Women
Source: Nutrients. 2022 Nov 2;14(21):4619. doi: 10.3390/nu14214619 (PMC9657450; doi:10.3390/nu14214619)
Supplement: Supplementary file 1 [file nutrients-14-04619-s001.zip › nutrients-1994472-supplementary.pdf]

Section S1. Yoghurt testing for pathogens and total aerobic counts after fermentation

The fermented samples were tested for the presence of the pathogens and total aerobic counts after fermentation. For the microbiological tests, 10 g of each sample was added to 90 ml of peptone water in a sterile stomacher bag, then mixed for two minutes (10^{-1} dilution). Suitable dilutions of the samples were prepared and plated on appropriate microbiological media. Nutrient agar (Oxoid, UK) was used for enumeration of total aerobic counts (Maturin and Peeler, 2001); the coliform test was performed using Violet Red Bile agar (VRBA) while Enterobacteriaceae were tested using Violet Red Bile Glucose agar (VRBGA) (Oxoid, UK) (AOAC. 1998). Baird-Parker Agar (BPA) (Oxoid, UK) was used for testing the presence of *Staphylococcus aureus* in the yoghurt samples. Three representative jars of yoghurt were analysed from each batch of coconut yoghurt with bioactives and placebo yoghurt. Following confirmation of the microbiological safety, the yoghurt samples were packed in a polystyrene chilly bin containing ice packs and transported overnight to the site of the human study.

Section S2. IL6 data

Table S1. Plasma IL6 (pg/ml) at baseline and different timepoint (min) after yoghurt consumption

	Placebo															
timepooint (min)	Participant 1	Participant 2	Participant 3	Participant 4	Participant 5	Participant 6	Participant 7	Participant 8	Participant 9	Participant 10	Participant 11	Participant 12	Participant 13	Participant 14	Participant 15	Participant 16
0	130.02	n.d	n.d	n.d	10.52	39.83	n.d	n.d	28.61	8.92	9.35	45.91	7.09	n.d	n.d	n.d
30	137	n.d	n.d	n.d	11.77	38.49	n.d	n.d	32.3	6.45	6.45	47.34	9.71	n.d	n.d	n.d
60	121.76	n.d	n.d	n.d	11.58	36.7	n.d	n.d	27.18	3.74	12.38	42.38	6.81	n.d	n.d	n.d
120	126.3	n.d	n.d	n.d	9.94	38.14	n.d	n.d	28.92	5.28	8.27	41.12	8.48	n.d	n.d	n.d
180	127.12	n.d	n.d	n.d	missing	40.81	n.d	n.d	31.93	10.94	8.9	41.96	14.24	n.d	n.d	n.d
240	144.64	n.d	n.d	n.d	11.58	43.92	n.d	n.d	28.76	11.26	13.09	42.47	15.39	n.d	n.d	n.d
	Bioactive yoghurt															
timepooint (min)	Participant 1	Participant 2	Participant 3	Participant 4	Participant 5	Participant 6	Participant 7	Participant 8	Participant 9	Participant 10	Participant 11	Participant 12	Participant 13	Participant 14	Participant 15	Participant 16
0	119.22	n.d	n.d	n.d	11.86	39.83	n.d	n.d	30.12	10.29	13.54	36.22	8.99	n.d	n.d	n.d
30	111.75	n.d	n.d	n.d	7.51	50.2	n.d	n.d	31.85	8.99	9.98	32.23	9.53	n.d	n.d	n.d
60	110.48	n.d	n.d	n.d	6.42	39.74	n.d	n.d	32.38	13.98	6.27	36.73	9.17	n.d	n.d	n.d
120	113.32	n.d	n.d	n.d	9.46	42.24	n.d	n.d	25.66	11.32	9.26	38.76	8.36	n.d	n.d	n.d
180	130.61	n.d	n.d	n.d	12.72	41.61	n.d	n.d	34.17	12.21	12.38	41.21	9.53	n.d	n.d	n.d
240	116.98	n.d	n.d	n.d	9.17	46.4	n.d	n.d	36.19	9.53	12.92	35.88	8.27	n.d	n.d	n.d

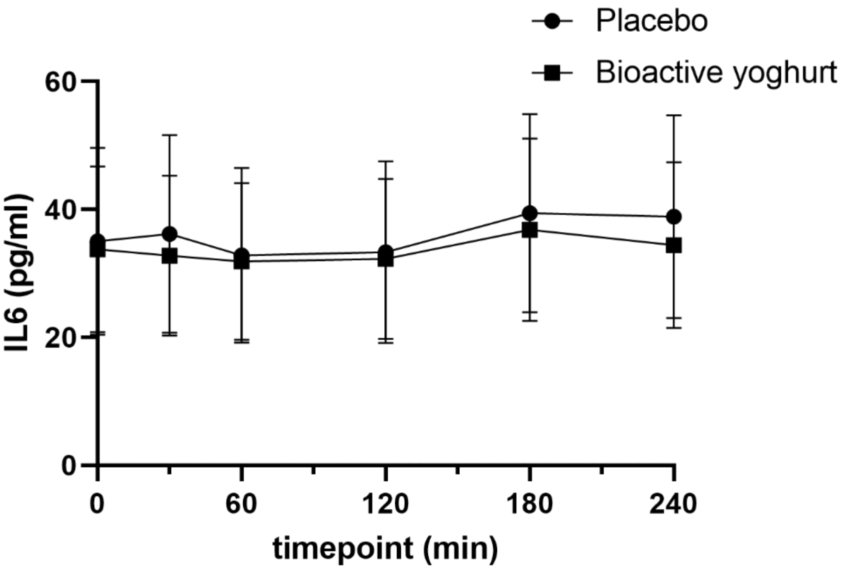

Plasma IL6. Data represented as mean and SEM

Figure S1 Uncle toby's breakfast bar nutrient label extracted from countdown NZ online website (11/10/2022)

Country of origin —

Made in Australia from at least 72% Australian ingredients

Ingredients +

Nutritional information —

Serving/pack: 4 Serving size: 65g

Nutrient	Per Serving[1]	Per 100g[1]	% Daily Intake Per Serving*
**Energy**	1090kJ	1680kJ	13%
**Protein**	5.2g	8g	10%
**Fat, total**	9.6g	14.8g	14%
Saturated	1.1g	1.7g	5%
**Carbohydrate**	33.9g	52.2g	11%
Sugars	7.5g	11.5g	8%
**Dietary Fibre**	7.3g	11.3g	24%
**Sodium**	13mg	21mg	<1%

* Percentage daily intakes are based on an average adult diet of 8700kJ. Your daily intakes may be higher or lower depending on your energy needs.

# All specified values are averages.

[1] Avg qty

Figure S2 Coconut sugar nutrient label from Matakana online store (11/10/2022)

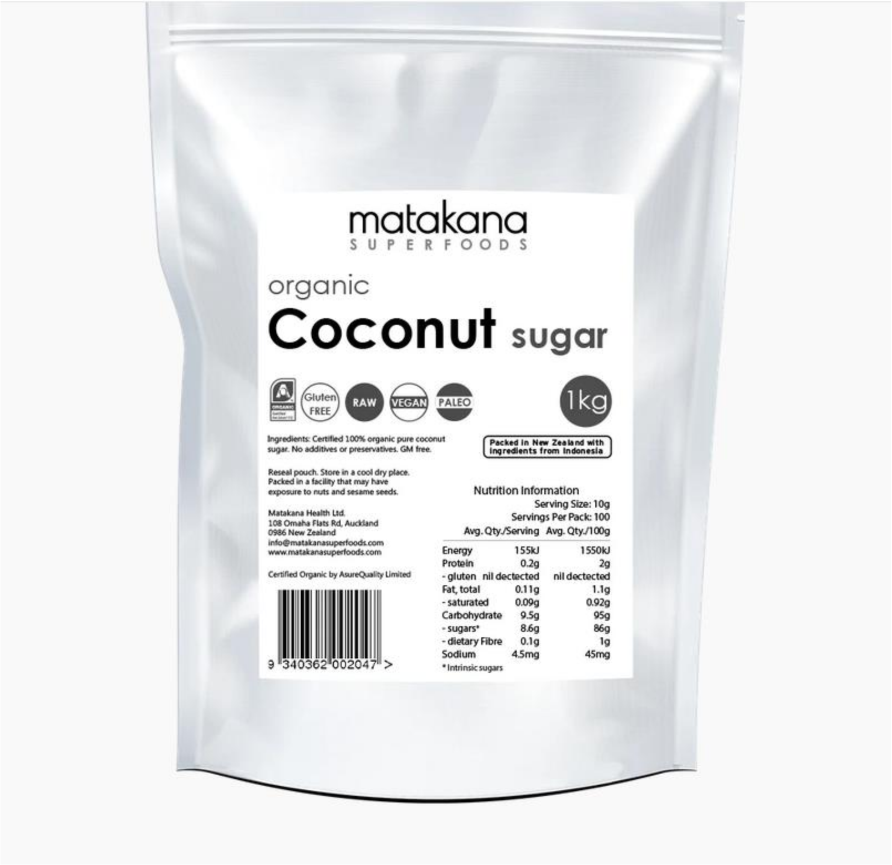

Table S2 Kara UHT Natural Coconut Cream nutrient summary from Foodworks 10 Version: 10.0.4266

Weight (g)	124
Protein (g)	3
Total fat (g)	30
Carbohydrate (g)	3
Sugars (g)	3
Water (g)	54
